# Interplay between noxious heat sensitivity and temporal summation magnitude in patients with fibromyalgia and long-term opioid use

**DOI:** 10.3389/fnins.2023.1275921

**Published:** 2023-10-12

**Authors:** Jason D. Bao, Morgan A. Rosser, Su Hyoun Park, Anne K. Baker, Katherine T. Martucci

**Affiliations:** ^1^Human Affect and Pain Neuroscience Laboratory, Department of Anesthesiology, Duke University Medical Center, Durham, NC, United States; ^2^Center for Translational Pain Medicine, Duke University Medical Center, Durham, NC, United States; ^3^Department of Anesthesiology, Biostatistics Group, Duke University School of Medicine, Durham, NC, United States

**Keywords:** temporal summation, sensitivity-adjusted temperature, opioids, fibromyalgia, chronic pain, opioid-related hyperalgesia, pain severity, central sensitization

## Abstract

**Introduction:**

In chronic pain conditions such as fibromyalgia (FM), pain amplification within the central nervous system, or “central sensitization,” may contribute to the development and maintenance of chronic pain. Chronic pain treatments include opioid therapy, and opioid therapy may maladaptively increase central sensitization, particularly in patients who take opioids long-term. However, it has remained unknown how central sensitization is impacted in patients who use opioids long-term.

**Methods:**

To investigate how long-term opioid therapy affects central sensitization, we used the validated measure of temporal summation. The temporal summation measurement consists of applying a series of noxious stimuli to a patient’s skin and then calculating changes in the patient’s pain rating to each stimulus. Using this measurement, we evaluated temporal summation in study participants with fibromyalgia who take opioids long-term (i.e., greater than 90 days duration; *n* = 24, opioid-FM). We compared opioid-FM responses to 2 control groups: participants with fibromyalgia who do not take opioids (*n* = 33, non-opioid FM), and healthy controls (*n* = 31). For the temporal summation measurement, we applied a series of 10 noxious heat stimuli (sensitivity-adjusted temperatures) to the ventral forearm (2s duration of each stimulus, applied once every 3 s). Additionally, we collected responses to standard pain and cognitive-affective questionnaires to assess pain severity and other factors.

**Results and discussion:**

Group differences in sensitivity-adjusted stimulus temperatures were observed, with only the non-opioid FM group requiring significantly lower stimulus temperatures (The opioid-FM group also required lower temperatures, but not significantly different from the control group). However, all 3 groups exhibited similar magnitudes of temporal summation. Across combined FM groups, temporal summation negatively correlated with pain severity (*r* = −0.31, *p* = 0.021). Within the opioid-FM group, higher pain sensitivity to heat (i.e., lower sensitivity-adjusted temperatures) showed a trend relationship with higher opioid dosage (*r* = −0.45, *p* = 0.036), potentially reflective of opioid-related hyperalgesia. Our findings also indicated that heightened pain severity may skew sensitivity-adjusted temporal summation, thereby limiting its utility for measuring central sensitization. Overall, in participants taking opioids, temporal summation may be influenced by hypersensitivity to heat pain, which appeared to vary with opioid dosage.

## 1. Introduction

While acute pain acts as a protective mechanism against tissue damage, progression to chronic pain can be debilitating. Characterized by plasticity of nociceptive pathways, chronic pain entails abnormal sensitization of the central nervous system (CNS) (i.e., central sensitization) and impaired pain modulatory systems, which together increase pain sensitivity (Ji et al., 2018).

Fibromyalgia (FM) is a chronic pain condition that involves increased sensitivity and pain widespread across the body (Clauw, 2014). Individuals with fibromyalgia demonstrate enhanced pain response to noxious and innocuous stimuli, altered pain circuits, and evidence of central sensitization (Gomez-Arguelles et al., 2018). Central sensitization can be reduced by administration of exogenous opioids, in line with opioids’ analgesic and hypoalgesic effects (Busse et al., 2018). However, in clinical trials, opioids often fail to reduce pain in individuals with fibromyalgia (Ngian et al., 2011; Goldenberg et al., 2016). Nonetheless, many patients with chronic pain continue to use opioids long-term. It remains an open question how long-term opioid use alters pain processing – specifically in terms of how opioids alter pain sensitivity and central sensitization.

Temporal summation is a validated procedure to evaluate the degree of central sensitization in humans based on physiological responses to repeated stimuli. It uses a series of repetitive noxious stimuli to calculate changes in pain response across the stimuli series (McMahon et al., 1993; Arendt-Nielsen et al., 1997). Repetitive heat stimuli at rates ≥0.33 Hz activate primary afferent nociceptors; the excessive input from these afferent fibers progressively increases perceived pain intensity (Vierck et al., 1997). Compared with healthy pain-free individuals, those with fibromyalgia demonstrate enhanced temporal summation (i.e., greater peak pain levels and lower pain thresholds) (Staud et al., 2001).

Chronic pain conditions are typically associated with enhanced magnitude of temporal summation. However, the impact of opioids on temporal summation is less clear. When acutely administered, opioids reduce temporal summation in both preclinical models of chronic pain (Lomas and Picker, 2005) and in clinical neuropathic pain patients (Suzan et al., 2016). Meanwhile, as shown in patients with fibromyalgia, temporal summation reductions occur with both placebo and acute treatments (Price et al., 2002). Thus, temporal summation diminution could result from drug administration or expectations.

Conversely, prolonged opioid use can lead to opioid-induced hyperalgesia (OIH), an increased sensitivity to painful stimuli (Lee et al., 2011). For example, after chronic pain patients receive 3 months of opioid therapy, heat pain thresholds decrease while temporal summation increases (Chen et al., 2009). Further, among patients with chronic back/neck pain who regularly use opioids, endogenous pain modulatory systems appear compromised (Martel et al., 2019). However, no published studies have tested temporal summation in individuals with fibromyalgia who take opioids long-term. Here, our objective in studying the degree of temporal summation was to clarify the impact of chronic opioid use on central sensitization. We further sought to determine the extent to which pain symptoms are influenced by duration, timing, and amount of opioid use.

In this study, we calculated temporal summation in participants with fibromyalgia who were taking opioids long-term (opioid-FM). We compared their temporal summation responses to healthy controls and to participants with fibromyalgia who were not taking opioids (non-opioid FM). In line with prior evidence of enhanced central sensitization in fibromyalgia, we expected that temporal summation would be increased in non-opioid FM compared to healthy controls. We hypothesized that opioid-FM participants would demonstrate the greatest temporal summation (i.e., enhanced central sensitization due to OIH-related symptoms). Lastly, because individuals with fibromyalgia also experience non-pain-related symptoms such as changes in affect/mood, we predicted that increased temporal summation in participants with fibromyalgia would correlate with greater cognitive-affective and clinical symptoms (e.g., anxiety, depression, pain severity, and negative affect).

## 2. Methods

### 2.1. Participants

Participants were recruited from the Durham, NC area. All data were collected from July 15, 2019 until May 1, 2022. The study included only female participants due to the greater prevalence of fibromyalgia in females and the need for sex-matched participant groups. All participants with fibromyalgia met the inclusion criteria of pain reported in 4 out of 5 body regions and self-reported average pain score of at least 4 out of 10 over the prior month. Additionally, all fibromyalgia participants met the 2016 revised American College of Rheumatology (ACR) criteria for fibromyalgia: widespread pain index [WPI] score ≥ 7 and symptom severity scale [SSS] score ≥ 5 or WPI score of 4–6 and SSS score ≥ 9; similar symptoms for at least 3 months; and pain attributable solely to fibromyalgia and no other disorder (Wolfe et al., 2016). Healthy controls were included only if they did not have chronic pain, take any pain or mood-altering medications, or experience any anxiety or depression at the time of the study. Participants were ineligible if they had any MRI contraindications.

All individuals with fibromyalgia and healthy controls signed a written informed consent indicating that they were willing to participate in the study, understood all study procedures, and could withdraw at any time. All study procedures were conducted in accordance with the Declaration of Helsinki, and approved by the Duke University Institutional Review Board.

### 2.2. Study procedures

#### 2.2.1. Sample size

**Figure 1:**
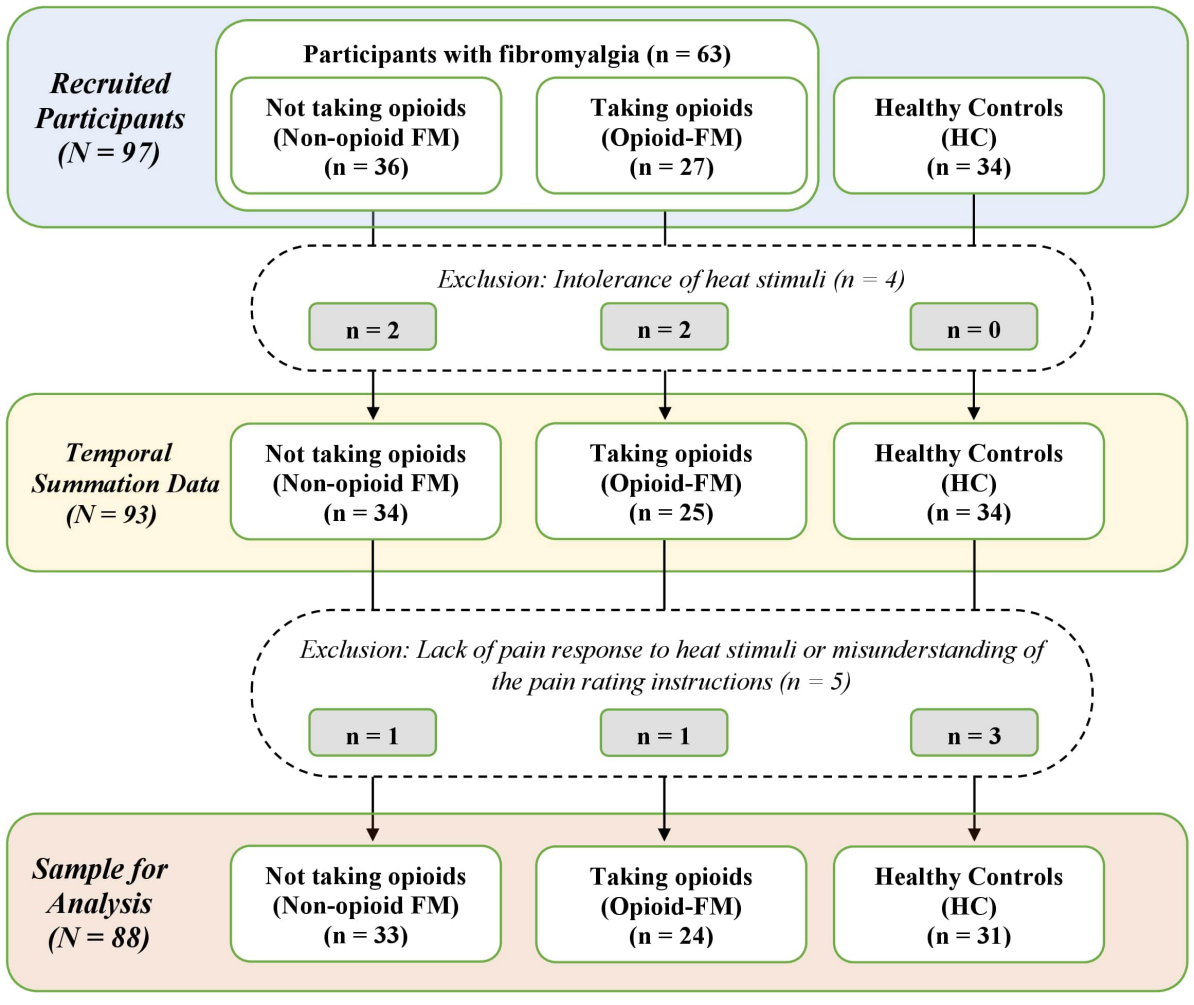
Flow chart of participant exclusions. Counts of participant datasets are shown for the stages of recruitment, data collection, and final sample used in the analysis. Non-opioid FM, participants with fibromyalgia who were not taking opioids; opioid-FM, participants with fibromyalgia who were taking opioids; HC, healthy controls.

We collected data from 97 participants: 36 participants with fibromyalgia who do not take opioids (non-opioid FM), 27 participants with fibromyalgia who take opioids (opioid-FM), and 34 healthy controls. Nine participants were excluded from the analysis: 4 were excluded due to their intolerance of the heat stimuli required for the temporal summation paradigm (non-opioid FM, *n* = 2; opioid-FM, *n* = 2), 4 were excluded due to a lack of pain response to the heat stimuli (healthy controls, *n* = 3; opioid-FM, *n* = 1), and 1 was excluded due to a misunderstanding of the pain rating instructions (non-opioid FM, *n* = 1). Thus, the final dataset used for analysis contained data for 88 participants, which included 33 non-opioid FM, 24 opioid-FM, and 31 healthy controls (Figure 1).

Prior to the analysis, and as part of a preregistered analysis plan, we ran a power analysis, using G*Power 3.1 software, on a sample size of 66 participants (22 per group). The power analysis showed that a one-way ANOVA to evaluate temporal summation in participants with fibromyalgia and healthy controls could detect a Cohen’s *F* effect size of 0.39 with ≥0.80 power at an alpha level of 0.05. Similarly, post-hoc Bonferroni *t*-tests could detect a Cohen’s *D* effect size of 1.01 with ≥0.80 power at an alpha level of 0.017. Given that prior literature reports an effect size of 1.47 for a 2-group comparison (Staud et al., 2001), our dataset was adequately powered to compare patients vs. healthy controls.

#### 2.2.2. Thermal pain threshold measurement

We conducted all quantitative sensory tests at Duke University in a private behavioral testing room at the Duke University Hospital. Similar to prior temporal summation protocols (Potvin et al., 2012; Staud et al., 2021), prior to the temporal summation test, we measured thermal pain thresholds. We administered thermal stimuli using a computer-connected Q-Sense thermode (Peltier-based device; 30 × 30-mm thermode surface area; Medoc Inc., Ramat Yishay, Israel). We placed the thermode on the right ventral forearm of each participant to test pain sensitivity. Then, we informed the participant that the temperature of the thermode would slowly change to higher temperatures. We asked participants to immediately report their pain level after each stimulus by using a visual analogue scale (VAS). The VAS is a validated scale for measuring experimental pain evoked by noxious heat stimuli (Price et al., 1983). The VAS used in our study was a double-sided slider. The side facing the participant had verbal anchors from “No pain at all” (0) to “Most painful sensation imaginable” (10), while the side facing the experimenter displayed a numeric scale of 0–10. For the thermal thresholding test, we increased the thermode temperature from 40°C to 47°C in 1-°C increments at a rate of 2°C/s. Using the participant’s pain ratings for each temperature, we determined the temperature that would evoke a pain rating of 5 on the VAS for each participant.

If a participant did not report a VAS rating of 5 during the first threshold test, we ran a second thresholding test on a slightly different part of the right forearm. For this second thresholding test, we increased the thermode’s temperature in smaller 0.2-°C increments, and tested temperatures between the 2 temperatures from the first threshold test that evoked pain ratings just below and above a rating of 5 on the VAS. Using pain ratings from the second thresholding test, we then chose the stimulus temperature that evoked a rating of 5, or closest to 5, on the VAS for the temporal summation paradigm.

#### 2.2.3. Experimental heat stimuli and temporal summation measurement

As described to participants, a “thermal tapping test” was then performed to measure temporal summation by using repeated “taps” of the thermode to the participant’s forearm. The thermode was preheated to each participant’s VAS = 5 temperature; the temperature remained constant throughout the test. Prior to the actual test procedure, 2 practice taps were used to show the participants the speed of the test, and how to rate their pain. As previous studies have not found consistent differences in heat pain sensitivity due to handedness (Long, 1994; Coghill et al., 2001; Sarlani et al., 2003) and to avoid habituation to heat stimuli during thresholding, the temporal summation protocol involved a thermal stimulus tap on the left ventral forearm every 3 s (2 s with the thermode touching the skin, 1 s with the thermode above the skin) for a total of 10 stimulus taps (Bosma et al., 2018). After each thermode tap, participants rated their pain on a case report form with 10 pre-drawn 10-cm lines. The case report form was designed as a paper version of the VAS, unnumbered, and with the number of centimeters indicating pain ratings on a 0–10 scale. After the testing session, the recorded pain ratings were measured with a ruler in centimeters and converted to a 1–100 scale from a 0–10 scale by multiplying by 10 (e.g., 0 ➔ 1, 0.1 ➔ 1, 1.1 ➔ 11). Conversion to this 1–100 scale allowed us to quantify temporal summation with both a difference and percentage calculation (see below). The converted data were then manually recorded onto electronic spreadsheets and double-checked for accuracy. We calibrated the temperature of the thermode before each test, using a built-in Medoc pre-test. The same Medoc Q-Sense thermode was used for all participants. Moreover, to limit the impact of expectation bias on pain ratings, we did not inform participants that the temperature would remain constant, or provide any indication that we expected summation to occur during the test.

For this study, we defined temporal summation as the calculated difference between the peak pain rating and baseline/initial pain rating (Staud et al., 2001). Others have used a percentage calculation of temporal summation to provide a relative measure of summation (Bosma et al., 2018). As the percentage calculation accounts for baseline pain rating variation, this calculation may potentially better portray the degree to which pain ratings change over time. As such, we also calculated temporal summation as the percent change from the baseline to peak pain rating (see Supplementary material).

### 2.3. Medication usage

Per our eligibility criteria, we required non-opioid FM participants to have <30 days of opioid use within their lifetimes, no opioid use within the 90 days prior to start of the study, and no opioid use for pain treatment during the study. We required opioid-FM participants to have taken opioid medications continuously for the 90 days prior to start of the study and for the duration of the study. Beyond these requirements, participants continued their normal use of medications during study participation.

As recorded during the study visits, opioid-FM participants were taking codeine (*n* = 1), hydrocodone (*n* = 2), hydrocodone/acetaminophen (*n* = 7), hydromorphone (*n* = 1), methadone (*n* = 1), morphine (*n* = 2), oxycodone (*n* = 1), oxycodone/acetaminophen (*n* = 1), tapentadol (*n* = 2), or tramadol (*n* = 9). Participants in both fibromyalgia groups were taking an assortment of other medications including nonsteroidal anti-inflammatory drugs (NSAIDs), serotonin-norepinephrine reuptake inhibitors (SNRIs), selective serotonin reuptake inhibitors (SSRIs), tricyclic antidepressants, other anxiolytics, antiepileptic drugs, and gamma-aminobutyric acid (GABA) analogues. Two FM participants took cannabidiol products, which have known analgesic effects (Mlost et al., 2020), 3 days before their respective study visits. In a post-hoc analysis, the exclusion of the data from these 2 participants did not significantly alter the group results, so their data were retained in the final analysis.

All healthy control participants had no history of chronic pain and were not taking any regularly prescribed medications at the time of the study. At the study visit, most of the healthy controls (*n* = 31) reported not taking any pain or mood-altering medications. Three healthy controls reported taking pain and/or mood-altering medications: one reported taking naproxen (~ 500–1,000 mg) for menstrual cramps on the day of the study visit, one reported taking ibuprofen one day prior to the study visit, and one reported taking zolpidem (5 mg) four days prior to the study visit. In a post-hoc analysis, the exclusion of the data from these participants did not significantly impact group results, so their data were retained in the final analysis.

### 2.4. Questionnaires

In addition to general demographic and medication questionnaires, all participants completed the following questionnaires: Beck Depression Inventory (BDI) (Beck et al., 1988), Behavioral Inhibition System/Behavioral Approach System (BIS/BAS) (Carver and White, 1994), Brief Pain Inventory (BPI) (Keller et al., 2004), Brief Symptom Inventory (BSI) (Derogatis and Melisaratos, 1983), Fibromyalgia Assessment Status (FAS) (Salaffi et al., 2009), Patient-Reported Outcomes Measurement Information System (PROMIS) Fatigue (Cella et al., 2010), Positive and Negative Affect Schedule (PANAS) (Watson et al., 1988), Profile of Mood States (POMS) (McNair et al., 1971), and State–Trait Anxiety Inventory (STAI-State, STAI-Trait) (Spielberger et al., 1970). Questionnaire data were collected and stored using a secure REDCap database. We used the data from the STAI-Trait, BDI, BPI, and PANAS questionnaires in analyses to test *a priori* hypotheses. We used data from the other questionnaires in our exploratory analyses.

### 2.5. Statistical analysis

A power analysis, study hypotheses, and all planned analyses were pre-registered on the Open Science Framework.^1^ The analyses were performed using R 4.1.3.

We used an omnibus one-way ANOVA across the 3 groups to analyze general characteristics of heat pain response, which included VAS = 5 temperatures, initial and peak stimulus ratings, and differences in temporal summation. Upon significant ANOVA results (*p* < 0.05), we performed additional post-hoc *t*-tests between groups (i.e., non-opioid FM vs. opioid-FM, healthy controls vs. non-opioid FM, and healthy controls vs. opioid-FM). We evaluated the ANOVA results at a Bonferroni-corrected threshold of *p* < 0.0167 to account for 3 comparisons of interest. Due to the potential impact of age, we ran an additional ANCOVA that adjusted for participant age while comparing initial pain ratings, peak pain ratings, and temporal summation.

Prior investigations have established a relationship between chronic pain and trait anxiety (Ruscheweyh et al., 2017), depressive symptoms (Grinberg et al., 2018; Overstreet et al., 2021), pain severity (Røsland et al., 2015), and negative affect (Staud et al., 2003). Therefore, we ran Spearman correlations to assess the relationships of temporal summation with our clinical/affective measures of trait anxiety (STAI-Trait), depression (BDI), average 24-h pain severity (BPI), and negative affect (PANAS, NAS subscale). Correlations between our selected clinical/affective measures resulted in 2 independent measures: (1) STAI-Trait, BDI, and NAS (*p* < 0.001) and (2) BPI pain severity (not correlated with other *a priori* variables). Thus, the correlations of temporal summation with clinical/affective measures were corrected for 2 independent comparisons, and determined to be statistically significant at *p* < 0.025.

### 2.6. Exploratory analyses of opioid use

We evaluated relationships of opioid use behaviors with pain sensitivity (as derived from the sensitivity-adjusted temperatures of each participant) and with temporal summation. For opioid use behaviors, we included opioid dosage (calculated in morphine milligram equivalents [MME]), duration of opioid use, and timing of last opioid dose (prior to the start of the study visit). We ran Spearman correlations using each of the 3 opioid use behavior variables vs. the 2 pain variables of VAS = 5 temperature and temporal summation. By testing correlations between the 3 opioid use behavior variables, we identified 2 independent measures: (1) duration of opioid use (not correlated with any other behavior variable) and (2) opioid dosage and timing of last opioid dose (*p* = 0.030; negative correlation). Therefore, we evaluated significance at a corrected threshold of *p* < 0.0125, correcting for 4 independent comparisons. Additionally, we ran exploratory analyses to identify potential effects of phase of opioid use (i.e., based on timing of opioid dose prior to study visit), other medication use, and other variables’ effects on temporal summation (see Supplementary material).

## 3. Results

### 3.1. Participants

The final analysis included data from 31 healthy controls, 33 non-opioid FM participants, and 24 opioid-FM participants. Most of the participants were right-handed (healthy controls: 94%; non-opioid FM: 94%; opioid-FM: 83%), white (healthy controls: 81%; non-opioid FM: 79%; opioid-FM: 83%), and had earned a college/university degree (healthy controls: 61%; non-opioid FM: 82%; opioid-FM: 75%). Most of the healthy controls and non-opioid FM participants were employed full-time (healthy controls: 58%; non-opioid FM: 58%); however, the opioid-FM group had a smaller percentage employed full-time (opioid-FM: 21%). Complete demographics are shown in Table 1.

**Table 1:** Participant demographics.

		HC	Non-opioid FM	Opioid-FM
Total number of participants		31	33	24
Right-handed		29	31	20
Hispanic or Latinx ethnicity		1	2	3
Self-identified race	Asian	3	1	0
	African American	2	5	2
	Caucasian	25	26	20
	American Indian/Alaskan/Pacific Islander	1	0	0
	Other	0	1	1
Employment status	Part-time employed	4	6	4
	Full-time employed	18	19	5
	Unemployed	4	5	6
	Retired	5	0	0
	Disabled	0	2	9
	Other	0	1	0
Education level	High school	0	1	2
	College/University	19	27	18
	Advanced degree	12	5	4

Two controls, two non-opioid FM participants, and four opioid-FM participants were left-handed. Two participants reported their race category as “other,” which refers to race other than all of the listed categories. One opioid-FM participant did not indicate race or ethnicity. “Part-time employed” and “unemployed” criteria included full-time students who worked part-time or were not employed. For education level, “High school” refers to “up to or through high school”; “College/University” refers to “up to or through college/university”; and “Advanced degree” refers to “any amount of education post college/university.” HC, healthy controls; non-opioid FM, participants with fibromyalgia who were not taking opioids; opioid-FM, participants with fibromyalgia who were taking opioids.

### 3.2. Opioid usage

Participants were asked to self-report their medication use on paper case report forms with assistance from the study experimenter. In the opioid-FM participant group, the median morphine milligram equivalent (MME) dosage per day was 15.53 mg, while the median duration of opioid use was 5 years. The distribution of opioid MME dosage per day was right-skewed, with all participants taking 5–40 mg/day except for 2 participants who were taking 85 mg/day and 90 mg/day, respectively. The distribution for opioid use duration was slightly less right-skewed, with opioid use ranging from 7 months to 10 years, with one outlier at 15 years opioid use duration. Across all participants with fibromyalgia (*n* = 57), 16 (28%) were taking NSAIDs, 19 (33%) were taking SNRIs, 10 (18%) were taking benzodiazepines, and 14 (25%) were taking GABA analogues. Further information about medication use for each group is presented in Table 2.

**Table 2:** Medication usage.

	HC (*n* = 31)	Non-opioid FM (*n* = 33)	Opioid-FM (*n* = 24)
Opioids	0	0	24
*Codeine*			1
*Hydrocodone*			2
*Hydrocodone/acetaminophen* (e.g.*, Norco*)			7
*Hydromorphone*			1
*Methadone*			1
*Morphine ER*			2
*Oxycodone*			1
*Oxycodone/acetaminophen* (e.g.*, Percocet*)			1
*Tapentadol* (e.g.*, Nucynta*)			2
*Tramadol*			9
NSAID (e.g., ibuprofen)	0*	8	8
Acetaminophen	0	5	4
SNRI (e.g., duloxetine)	0	10	9
SSRI (e.g., fluoxetine)	0	6	3
Tricyclic Antidepressant (e.g., amitriptyline)	0	1	1
Other Anxiolytic (e.g., buspirone)	0	1	6
Antiepileptic (e.g., topiramate)	0	4	7
Triptans (e.g., sumatriptan)	0	5	2
SARI (e.g., trazodone)	0	2	5
NDRI (e.g., methylphenidate, bupropion)	0	5	3
Benzodiazepine (e.g., clonazepam)	0	7	3
Benzodiazepine-like (e.g., eszopiclone)	0*	1	3
Muscle Relaxant (e.g., cyclobenzaprine)	0	8	12
GABA Analogue (e.g., gabapentin)	0	5	9
CBD (e.g., oil, tincture)	0	1	2
*Taking no medications*	31	3	0

The number of healthy controls, non-opioid FM, and opioid-FM who were taking each class of medication is shown. *See exceptions in section 2.3 of text. HC, healthy controls; non-opioid FM, participants with fibromyalgia who were not taking opioids; opioid-FM, participants with fibromyalgia who were taking opioids; NSAID, nonsteroidal anti-inflammatory drug; SNRI, serotonin and noradrenergic reuptake inhibitor; SSRI, selective serotonin reuptake inhibitor; SARI, serotonin antagonist and reuptake inhibitor; NDRI, norepinephrine-dopamine reuptake inhibitor; GABA, gamma-aminobutyric acid; CBD, cannabidiol.

### 3.3. Clinical and psychological measures

Questionnaire data revealed significant differences between the healthy controls and fibromyalgia groups. Compared to healthy controls, the fibromyalgia groups demonstrated more pain areas, greater fatigue, higher negative affect, worse mood, and higher anxiety (Table 3). Despite our efforts to recruit similar age ranges among the groups, we identified a significant interaction between age and group [*F*_(2, 85)_ = 5.965, *p* = 0.004]. Post-hoc *t*-tests revealed that the non-opioid FM cohort (37.70 ± 13.32 years) was significantly younger than the opioid-FM cohort (48.96 ± 9.70 years).

**Table 3:** Clinical, psychological, and behavioral variables.

	HC (*n* = 31)		Non-opioid FM (*n* = 33)		Opioid-FM (*n* = 24)		ANOVA	FM *t*-test
	*n*	Mea*n* ± sd	*n*	Mea*n* ± sd	*n*	Mea*n* ± sd	*p*-Value	*p*-Value
Age	31	44.1 ± 13.1	33	37.7 ± 13.3	24	49.0 ± 9.7	0.004	0.003*
Depression (BDI)	30	2.4 ± 3.6	33	18.4 ± 9.6	24	17.6 ± 10.5	< 0.001	0.942
Behavioral inhibition system (BIS)	31	19.7 ± 3.2	33	21.1 ± 3.7	24	21 ± 4.7	0.302	0.999
Behavioral approach system (BAS)	31	39.7 ± 4.8	33	37.9 ± 5.6	24	38.7 ± 5.5	0.400	0.844
Pain severity (BPI)	–	–	31	5.3 ± 1.2	24	5.8 ± 1.3	–	0.197
Pain interference (BPI)	–	–	31	6.7 ± 2.0	24	6.3 ± 2.5	–	0.502
Global severity index (BSI)	30	2.5 ± 2.9	33	19.6 ± 10.3	24	16.3 ± 13.6	< 0.001	0.404
Total pain areas (FAS)	30	0.5 ± 0.9	33	13.1 ± 3.4	24	12.7 ± 4.1	< 0.001	0.864
Cognitive (FAS)	30	1.3 ± 2	33	9.7 ± 1.3	24	8.9 ± 1.9	< 0.001	0.252
Comorbid (FAS)	30	0.2 ± 0.5	33	2.2 ± 0.9	24	2.1 ± 0.9	< 0.001	0.891
Fatigue (PROMIS)	30	43.1 ± 6.8	33	67.1 ± 6.3	24	65.3 ± 8.0	< 0.001	0.597
Positive affect (PANAS)	30	36.3 ± 6.1	33	23.7 ± 7.2	24	26 ± 7.6	< 0.001	0.412
Negative affect (PANAS)	20	13.9 ± 3.9	33	21.2 ± 6.1	24	19.5 ± 7.5	< 0.001	0.540
Tension (POMS)	30	1.1 ± 1.4	33	4.4 ± 3.9	24	4.3 ± 4.5	< 0.001	0.981
Depression (POMS)	30	0.5 ± 1.2	33	2.9 ± 3.8	24	3.2 ± 5.0	0.008	0.970
Anger (POMS)	30	0.4 ± 0.9	33	2.2 ± 3.6	24	2 ± 3.3	0.024	0.973
Fatigue (POMS)	30	1.8 ± 1.6	33	10.4 ± 4.3	24	11.3 ± 4.8	< 0.001	0.648
Confusion (POMS)	30	2.5 ± 1.1	33	4.5 ± 3.1	24	5.0 ± 3.4	0.003	0.768
Vigor (POMS)	30	8.6 ± 4.2	33	3 ± 3.2	24	2.5 ± 2.1	< 0.001	0.818
Total mood disturbance (POMS)	30	−2.1 ± 6.1	33	21.5 ± 15.6	24	23.3 ± 19.4	< 0.001	0.884
State anxiety (STAI)	29	28.8 ± 6.9	32	39.8 ± 9.4	24	39.9 ± 12.4	< 0.001	0.999
Trait anxiety (STAI)	30	30.8 ± 7.2	32	44.4 ± 10	21	42.9 ± 10.2	< 0.001	0.811

Participant counts (*n*) for each measure may differ from the total number of participants because some participants did not complete all questionnaires. Healthy controls, for example, did not complete the BPI questionnaire to assess pain severity and impact on daily function, due to their eligibility requirement for no history of chronic pain. HC, healthy controls; non-opioid FM, fibromyalgia participants who were not taking opioids; opioid-FM, fibromyalgia participants who were taking opioids; BDI, beck depression inventory; BIS/BAS, behavioral inhibition system/behavioral approach system; BPI, brief pain inventory; BSI, brief symptom inventory; FAS, fibromyalgia assessment status; PROMIS, patient-reported outcomes measurement information system; PANAS, positive and negative affect schedule; POMS, profile of mood states; STAI, state–trait anxiety inventory; sd, standard deviation. *p*-values for the one-way analysis of variances (ANOVA) across the 3 groups are reported for significance of group effect. *Post-hoc t*-test *p*-values are reported for comparison between the non-opioid FM and opioid-FM groups; significance was evaluated at a value of *p* < 0.0125, corrected for multiple comparisons. *p < 0.0125.

As shown by post-hoc *t*-tests, the questionnaire responses were not significantly different between the 2 fibromyalgia groups. Both fibromyalgia groups reported similar number of pain areas (FAS), similar level of pain severity, and similar level of pain interference (BPI). Participants in the non-opioid FM group reported 5 to 19 (maximum) body areas with pain, average 24-h pain severity (5.3/10), and average pain interference (6.7/10). Likewise, opioid-FM participants reported 4 to 19 body areas with pain, average 24-h pain severity (5.8/10), and average pain interference (6.3/10). Thus, as validated by these reported pain variables, our sample of participants with fibromyalgia had widespread distribution and daily significance of pain.

### 3.4. Temporal summation characteristics across groups

In order to reduce variability due to individual differences in thermal sensitivity, we calibrated the stimulus temperature for each individual that evoked a pain rating of 5 on the VAS. The average individually calibrated stimulus temperature for each group was 46.8 ± 1.5°C for healthy controls, 45.4 ± 2.2°C for non-opioid FM, and 45.7 ± 2.0°C for opioid-FM [*F*_(2, 85)_ = 4.505, *p* = 0.014]. Although the participants in both fibromyalgia groups required lower temperatures to evoke a pain rating of 5 on the VAS, post-hoc tests identified significant group differences in the VAS = 5 temperature between only the healthy controls and non-opioid FM group (*p* = 0.005). Temperatures required to evoke a VAS of 5 were not significantly different for the healthy controls vs. opioid-FM group (*p* = 0.039) nor the non-opioid FM group vs. opioid-FM group (*p* = 0.333).

**Figure 2:**
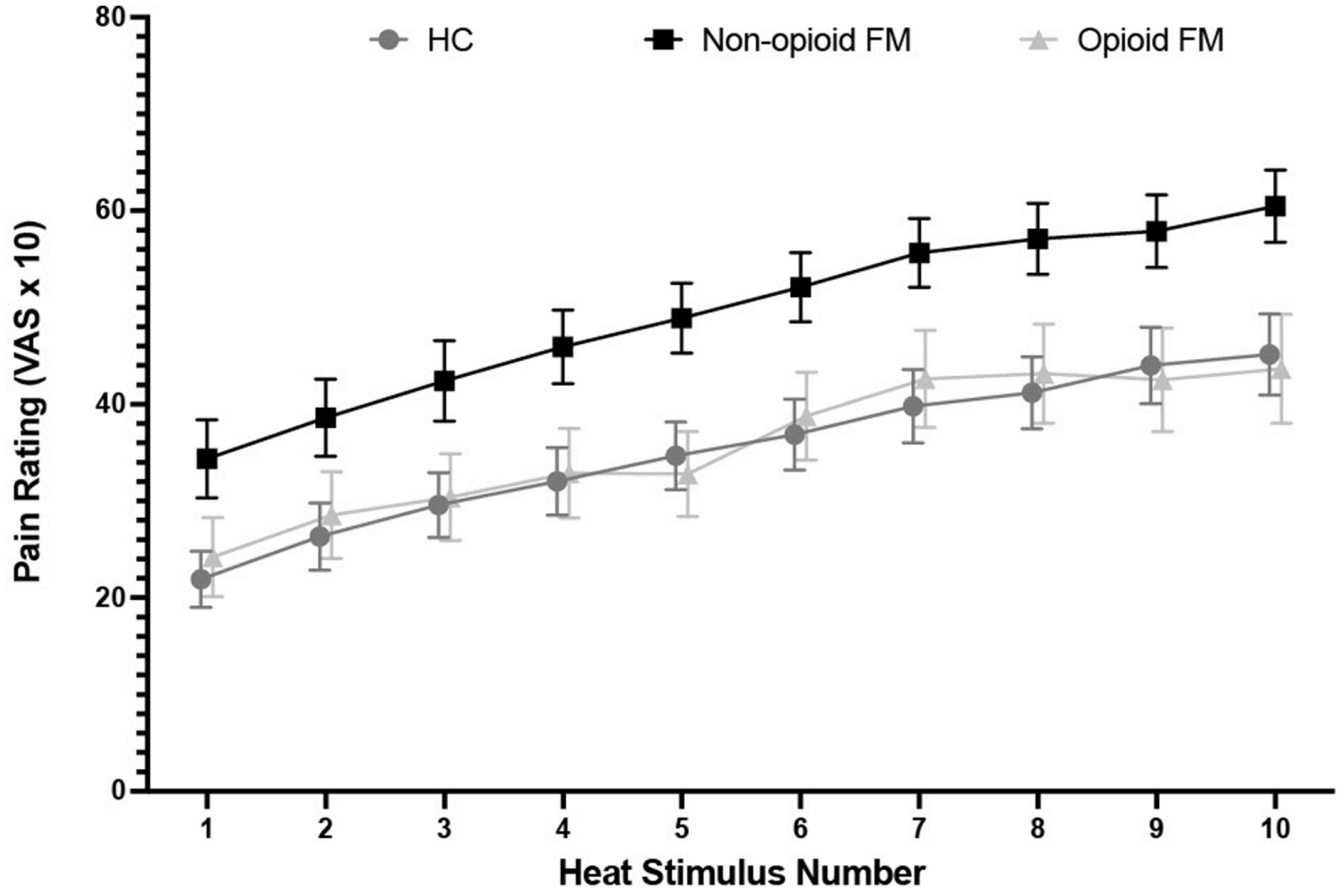
Pain rating distribution across 10 repeated heat stimuli by group. For the temporal summation test, participants received a stimulus tap for 2 s at a frequency of 0.33 Hz with an interstimulus interval of 1 s, and provided pain ratings after each stimulus. Stimulus temperature was calibrated to a VAS = 5 temperature that was determined for each individual participant before the experiment began. Error bars indicate standard error. HC, healthy controls; non-opioid FM, participants with fibromyalgia who were not taking opioids; opioid-FM, participants with fibromyalgia who were taking opioids.

Across the 10 stimuli of the temporal summation test, pain ratings increased from 34.21 ± 23.30 to 63.39 ± 20.24 in the non-opioid FM group, from 24.13 ± 19.98 to 49.25 ± 25.12 in the opioid-FM group, and from 21.81 ± 16.16 to 48.61 ± 21.51 in healthy controls (Figure 2). Overall, within each group, peak pain ratings were significantly higher than initial pain ratings [healthy controls: t_(30)_ = 8.84, *p* < 0.001; non-opioid FM: t_(32)_ = 10.578, *p* < 0.001; opioid-FM: t_(23)_ = 6.124, *p* < 0.001]. This indicated an overall increase in pain across repetitive heat stimuli, as expected with temporal summation.

**Figure 3:**
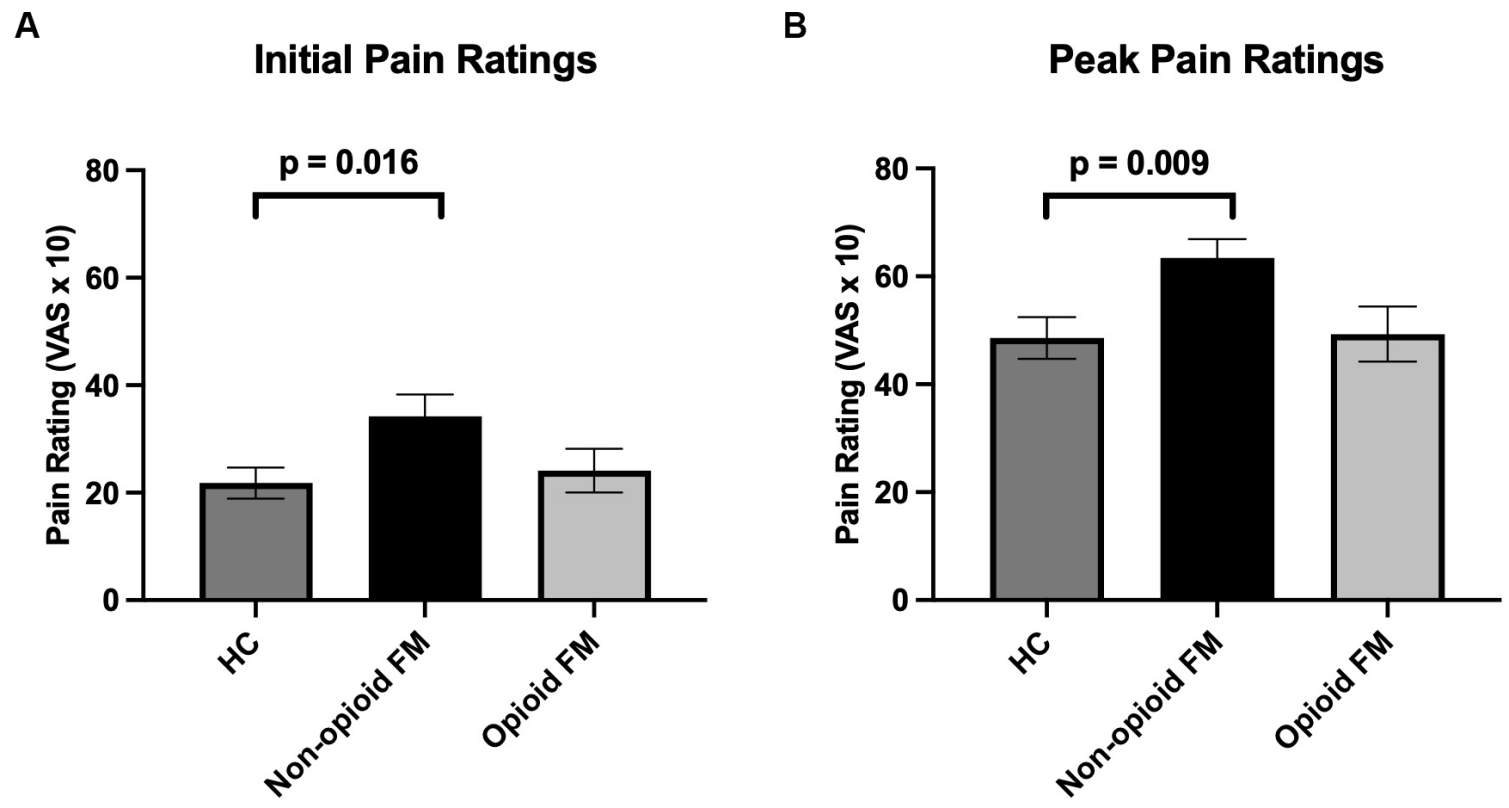
Mean initial and peak pain ratings across all participant groups. Pain ratings were analyzed separately for initial pain ratings (i.e., the pain rating after the first stimulus in the temporal summation stimulus series) and peak pain ratings (i.e., the highest reported pain rating to any stimulus from the 2nd to 10th stimulus in the temporal summation stimulus series). **(A)** Initial pain ratings were significantly different across groups, and post-hoc testing revealed that this difference was primarily due to significantly higher initial pain ratings in the non-opioid FM group compared to healthy controls. **(B)** For peak pain ratings, non-opioid FM participants exhibited significantly higher peak pain ratings compared to healthy controls. Error bars indicate standard error. HC, healthy controls; non-opioid FM, participants with fibromyalgia who were not taking opioids; opioid-FM, participants with fibromyalgia who were taking opioids.

Across the 3 groups, significantly different responses were observed for both initial and peak pain ratings [initial: *F*_(2, 85)_ = 3.399, *p* = 0.038; peak: F_(2, 85)_ = 4.448, *p* = 0.015]. As indicated by post-hoc two-sample *t*-tests (Bonferroni corrected *p*-value, *p* < 0.0167), initial and peak pain ratings were significantly higher in the non-opioid FM group compared to the healthy controls [initial: t_(62)_ = 2.464, *p* = 0.016; peak: t_(62)_ = 2.832, *p* = 0.009] (Figure 3). However, initial and peak pain ratings were not significantly different in the opioid-FM group compared to the healthy controls (initial: *p* = 0.333; peak: *p* = 0.333) nor compared to the non-opioid FM group (initial: *p* = 0.065; peak: *p* = 0.019). Furthermore, as indicated by the ANCOVA with age as a covariate, initial and peak pain ratings were significantly different across groups [initial: *F*_(2, 85)_ = 3.456, *p* = 0.036; peak: F_(2, 85)_ = 4.613, *p* = 0.013].

**Figure 4:**
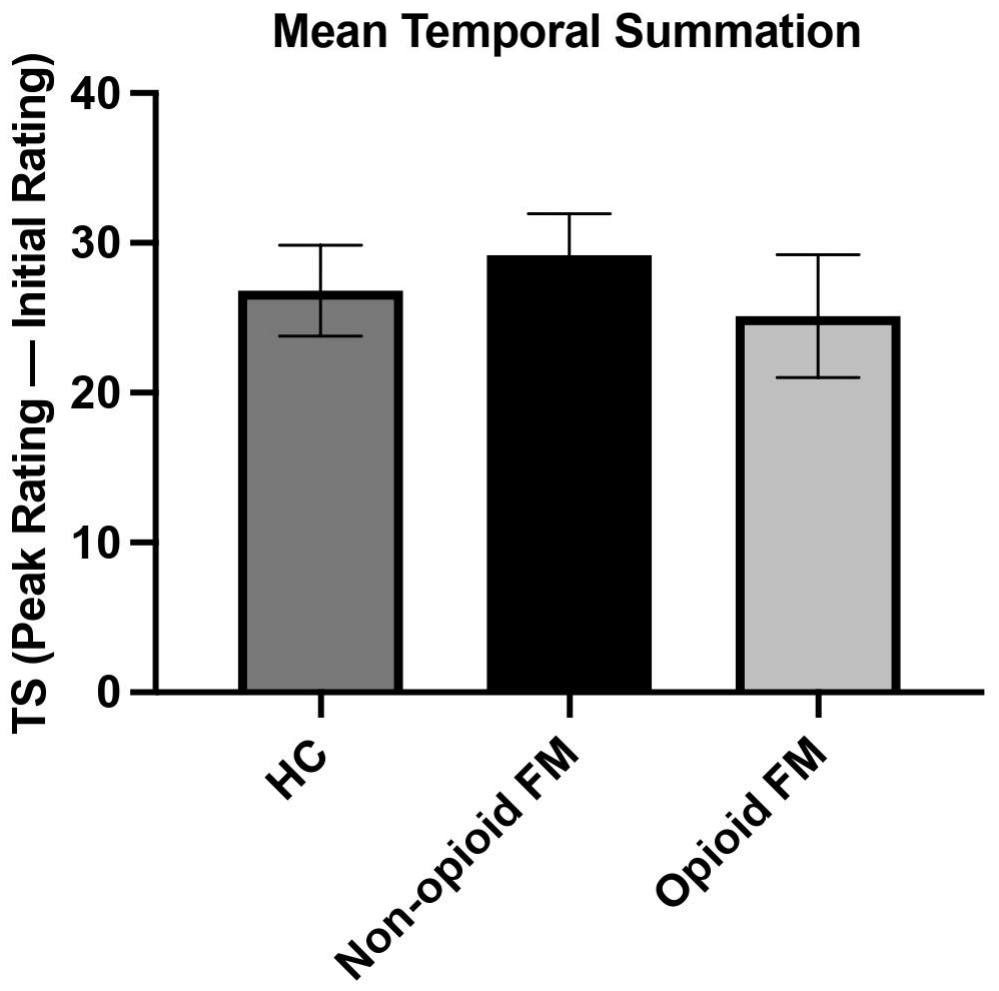
Mean temporal summation across all participant groups. For each participant group, temporal summation was calculated as the difference between peak and initial pain ratings. The average temporal summation of each group was as follows: healthy controls 26.81 ± 16.89; non-opioid FM 29.18 ± 15.85; and opioid-FM 25.13 ± 20.10. One participant in each group did not experience summation (i.e., these 3 participants did not report increasing pain responses as the series of 10 stimuli progressed). Temporal summation did not differ significantly between the participant groups. Error bars indicate standard error. HC, healthy controls; non-opioid FM, participants with fibromyalgia who were not taking opioids; opioid-FM, participants with fibromyalgia who were taking opioids; TS, temporal summation.

Temporal summation was not significantly different across all 3 groups [F_(2, 85)_ = 0.390, *p* = 0.678] (Figure 4). A post-hoc ANCOVA with age as a covariate confirmed no significant difference in temporal summation across all 3 groups [F_(2, 85)_ = 0.388, *p* = 0.680]. Finally, an additional post-hoc analysis that excluded participants who did not exhibit temporal summation to repeated stimuli (one healthy control, one non-opioid FM, and one opioid-FM) also showed no significant differences in temporal summation [*F*_(2, 82)_ = 0.369, *p* = 0.692].

### 3.5. Explored clinical and psychological variables with temporal summation

Measured across all participants, STAI-trait anxiety (*r* = 0.06, *p* = 0.606, *n* = 83), BDI depression (*r* = 0.17, *p* = 0.110, *n* = 87), and PANAS negative affect (*r* = 0.04, *p* = 0.692, *n* = 87) were not significantly correlated with temporal summation (Table 4).

**Table 4:** Clinical and psychological associations with temporal summation.

Variable	TS – All Participants (*N* = 88)		TS – FM (*n* = 57)		TS – Non-opioid FM (*n* = 33)		TS – Opioid-FM (*n* = 24)	
	Rho	*P*-Value	Rho	*P*-Value	Rho	*P*-Value	Rho	*P*-Value
Trait anxiety (STAI)	0.06	0.606	0.06	0.675	−0.04	0.816	0.12	0.605
Depression (BDI)	0.17	0.110	0.16	0.243	0.13	0.473	0.16	0.470
Pain severity (BPI)	–	–	−0.31*	0.021	−0.20	0.280	−0.45	0.029
Negative affect (PANAS)	0.04	0.692	−0.10	0.470	−0.12	0.512	−0.25	0.239

Spearman correlations across all participants compared clinical, psychological, and behavioral variables with temporal summation. Correlations were also assessed within each fibromyalgia subgroup (non-opioid FM and opioid-FM participants). Comparisons differed in the number of data values (± 5), due to some incomplete questionnaire forms during the study visit. A significant correlation between pain severity and reduced temporal summation was observed within the combined fibromyalgia cohort. Healthy controls did not complete the BPI questionnaire. Significance was observed at a Bonferroni-corrected *p*-value <0.025. *p < 0.025. FM, combined fibromyalgia cohort; TS, temporal summation; non-opioid FM, participants with fibromyalgia who were not taking opioids; opioid-FM, participants with fibromyalgia who were taking opioids.

**Figure 5:**
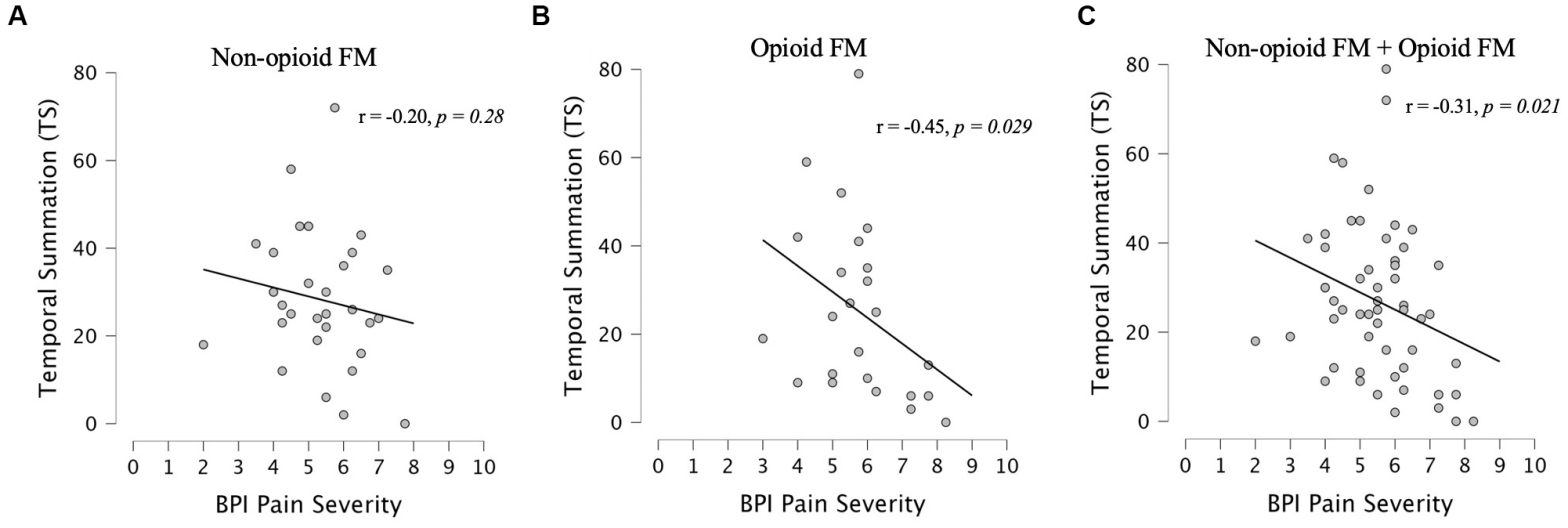
Correlations between pain severity and temporal summation. Spearman correlations between BPI pain severity and temporal summation were assessed within each fibromyalgia group [non-opioid FM **(A)** and opioid-FM **(B)**] and within the combined cohort of participants with fibromyalgia **(C)**. Healthy controls did not complete the BPI questionnaire. At a Bonferroni-corrected value of *p* <0.025, a significant negative correlation between BPI pain severity and temporal summation was observed in the combined cohort of fibromyalgia participants (*r* = −0.31, *p* = 0.021) – this indicated less temporal summation among participants with higher pain severity. Similar trends were observed in each fibromyalgia subgroup, but did not reach significance. Non-opioid FM, participants with fibromyalgia who were not taking opioids; opioid-FM, participants with fibromyalgia who were taking opioids.

Measured across fibromyalgia groups (non-opioid FM and opioid-FM), average 24-h pain severity (BPI) was significantly negatively correlated with temporal summation (*r* = −0.31, *p* = 0.021, *n* = 55) (Figure 5). Pain severity was correlated with initial pain ratings, but did not survive Bonferroni correction (*r* = 0.23, *p* = 0.043, *n* = 55). We also evaluated the relationship between temporal summation and other variables (i.e., age, race, state anxiety, positive affect, total mood disturbance, average pain interference, total pain areas, fatigue, and global severity index) across all participants (see Supplementary material).

### 3.6. Explored relationships between opioid use and temporal summation

Within the opioid-FM group, after excluding one participant who did not demonstrate temporal summation (*n* = 23), temporal summation was not significantly correlated with opioid MME dosage, duration of opioid use, or timing of last opioid dose (dosage: *r* = 0.19, *p* = 0.391, *n* = 22; duration of use: *r* = 0.26, *p* = 0.251, *n* = 21; timing: *r* = −0.14, *p* = 0.533, *n* = 21). Opioid dosage was related to sensitivity-adjusted (VAS = 5) temperature. Specifically, higher opioid dosage correlated with increased sensitivity to heat stimuli (*r* = −0.45, *p* = 0.036, *n* = 22); however, this relationship did not survive Bonferroni correction at a value of *p* <0.0125. All correlation results are shown in Table 5. We also compared temporal summation and each opioid variable within 2 subgroups: early- and late-phase opioid-FM participants (see Supplementary material).

**Table 5:** Opioid use characteristics and pain response.

Opioid variable	VAS = 5 Temperature			Temporal summation		
	*n*	Rho	*P*-value	*n*	Rho	*P*-value
Opioid dosage	22	−0.45	0.036	22	0.19	0.391
Duration of opioid use	21	0.01	0.966	21	0.26	0.251
Timing of last dose	21	−0.003	0.989	21	−0.14	0.533

Patterns of opioid use were evaluated against the experimental VAS = 5 temperature and temporal summation across all opioid-FM participants. One participant did not exhibit temporal summation; one participant did not report daily dosage; 2 participants did not report duration of use; and one participant did not report timing of last dose. Spearman correlations were assessed for significance at a Bonferroni-corrected threshold of *p* < 0.0125.

## 4. Discussion

This was the first study to assess the experience of temporal summation in individuals with fibromyalgia who take long-term opioids. Temporal summation was evoked by administering repetitive stimuli at sensitivity-calibrated temperatures. As indicated by predetermined sensitivity-adjusted temperatures, the non-opioid FM group exhibited greater pain sensitivity compared to healthy controls. Meanwhile, healthy control and opioid-FM groups demonstrated comparable pain sensitivity. Notably, all groups demonstrated similar temporal summation magnitude. Thus, our hypotheses that non-opioid FM and opioid-FM participants would demonstrate greater heat pain sensitivity and temporal summation than healthy controls were not confirmed.

Importantly, from our correlation analyses across fibromyalgia groups, we identified a trend toward lower temporal summation in participants who reported higher pain severity (Of note, this finding was contrary to our hypothesized positive correlation between pain severity and temporal summation). Further, suggestive of opioid-related hyperalgesia, in the opioid-FM group, higher pain sensitivity was related to higher opioid dosage. As indicated by our results, chronic pain severity modulates sensitivity-adjusted measurement of temporal summation. Additionally, chronic opioid dosage may affect the extent to which chronic opioid use modifies central sensitization.

### 4.1. Abnormal pain processing in participants with fibromyalgia

Compared to opioid-FM and healthy control groups, the non-opioid FM group demonstrated higher initial pain ratings and higher peak pain ratings. Notably, the higher pain ratings occurred in the non-opioid FM group despite significantly lower sensitivity-adjusted stimulus temperatures.

All groups exhibited similar magnitudes of temporal summation. Consistently, with across-group comparisons using a percentage calculation for temporal summation, all groups still exhibited similar temporal summation magnitudes (see Supplementary Results 2.1). While we expected to identify greater temporal summation in our FM groups, our results mirror prior evidence for similar pain responses among healthy controls and individuals with fibromyalgia (Potvin et al., 2012; Bosma et al., 2016; Staud et al., 2021). Notably, these studies used repeated heat stimuli at sensitivity-adjusted temperatures, similar to the procedures we used in this study.

The opioid-FM group demonstrated VAS = 5 temperatures and temporal summation that were analogous to healthy controls. Pain characteristics of the opioid-FM group provide some support for potential analgesic effects of opioid agonists (Stein, 2016). Indeed, these findings parallel prior findings of reduced temporal summation in individuals with chronic pain who take clinically administered morphine sulfate (Price et al., 1983) or oxycodone (Suzan et al., 2013). These earlier studies, together with our findings in FM patients with chronic opioid use, suggest that opioid use may reduce some aspects of pain response in individuals with fibromyalgia.

Our opioid-FM and non-opioid FM groups had similar VAS = 5 temperatures and temporal summation. Compared to opioid-FM participants, we observed only a trend toward higher peak pain ratings among non-opioid FM. Thus, while not modulating pain sensitivity (i.e., individually sensitivity-determined temperatures), opioid use may modulate the upper bounds of central sensitization (i.e., peak pain ratings).

Distinct mechanisms may contribute to processing afferent pain signals at classically defined innocuous temperatures (i.e., < 45°C) vs. classically defined noxious temperatures (≥ 45°C). Prior studies of FM have identified greater temporal summation at fixed temperatures of 49.5–52°C (Staud et al., 2001; Price et al., 2002). Therefore, in our supplementary analyses, we divided all participants into subgroups based on VAS = 5 temperature. After excluding participants with sensitivity-adjusted temperatures below 45°C, we still identified no group differences in temporal summation (see Supplementary material). The conflicting results may be due to the lower intensity stimuli used in our study.

Finally, because the stimulus number that evoked a “peak” pain rating varied by participant, it was challenging to accurately compare summation rates using the 1st, 5th, and 10th stimuli (see Supplementary material). Nonetheless, pain-rating fluctuations across repeatedly administered stimuli provide rich datasets of information. Future efforts should take advantage of these stochastic data to better quantify temporal summation using computational modeling.

### 4.2. Clinical and demographic factors associated with temporal summation

In contrast with our initial hypothesis and prior findings (Castelo-Branco et al., 2022), we observed a negative relationship between temporal summation and pain severity among both our non-opioid FM and opioid-FM groups. While this correlation only reached significance across combined FM groups, similar trends were evident in each FM group, with the opioid-FM group driving the relationship. Thus, regardless of opioid use, patients with the highest reported pain severity exhibited the least temporal summation. Due to the nature of VAS ratings, peak pain ratings may have been influenced by a ceiling effect, thereby decreasing calculated summation in individuals who reported higher pain ratings to the stimuli. Thus, even though we attempted to use personalized (i.e., sensitivity-adjusted) temperatures to measure temporal summation, temporal summation may be methodologically limited in patients who report high pain severity.

Of note, although we calibrated the stimulus temperature for each participant based on her VAS = 5 rating, many FM participants rated their pain to the initial temporal summation stimulus as VAS > 5. Pain ratings can be increased by psychological states such as expectation (Koyama et al., 2005), depression (Gorczyca et al., 2013), and state anxiety (Lacourt et al., 2014). While we did not assess expectations prior to the temporal summation test, neither depression nor state anxiety significantly correlated with temporal summation among our participant cohort. By including pain-free control groups with depression and/or elevated levels of state anxiety, future investigations may better differentiate influences of cognitive and affective states on pain sensitivity and temporal summation. Moreover, while our thresholding procedures (to determine VAS = 5 temperatures) were similar to those used by others (Chen et al., 2009; Staud et al., 2014; Bosma et al., 2016), increased initial ratings to VAS = 5 temperatures during temporal summation testing may have resulted from procedural differences between thresholding (i.e., 5 s stimulus duration) vs. the temporal summation test (i.e., 2 s stimulus duration).

### 4.3. Pain rating patterns potentially reflect opioid-related hyperalgesia

When receiving long-term opioid therapy, chronic pain patients show enhanced temporal summation and exacerbated hyperalgesia (Chen et al., 2009; Compton et al., 2020). While we did not identify enhanced temporal summation in our opioid-FM group, opioid dosage was negatively correlated with timing of the last opioid dose (i.e., higher dosage correlated with more recent last opioid dose). This correlation potentially reflects greater susceptibility to pain during withdrawal, which may relate to more frequent opioid use. We noted trend relationships of (1) greater heat pain sensitivity (i.e., lower VAS = 5 temperature) with higher opioid dosage, and (2) greater temporal summation (when evaluated as a percentage change) with less recent last opioid dose (see Supplementary material). As suggested by these findings, pain hypersensitivity and development of opioid-related hyperalgesia may be most relevant at more frequent and higher opioid dosage.

### 4.4. Limitations

Our results should be considered with some limitations. First, all opioid-FM participants were taking multiple (i.e., opioid and non-opioid) medications and a variety of opioid formulations (i.e., immediate-release and/or extended-release). Such use of other medications with distinct analgesic and psychoactive profiles could differentially contribute to temporal summation changes; however, we were underpowered to analyze the effects of unique medications and/or different combinations of medications (see Supplementary material). Second, for our assessments of temporal summation, our ability to accurately identify sensitivity-calibrated temperatures could have been impacted by heightened pain anticipation and motivational deficits that occur in fibromyalgia. Among individuals with chronic pain, differences in pain anticipation and expectations can alter pain experience (Brown et al., 2014; Lindheimer et al., 2019). Individuals with fibromyalgia exhibit altered reward systems and motivation response (Loggia et al., 2014; Martucci et al., 2018). Therefore, future investigations of temporal summation should assess expectations and motivation directly. Third, due to the greater prevalence of fibromyalgia in females, our comparison focused on comparing temporal summation and heat pain sensitivity among females only. It is possible that sex-based pain perception and sensitivity differences could impact temporal summation in fibromyalgia (Ruschak et al., 2023). Lastly, while 3 different female experimenters collected data for the present study, they were trained together using the same instructional scripts and protocol; we did not detect significant differences in temporal summation between datasets collected by each experimenter (see Supplementary material).

## 5. Conclusion

Our study presents the first evaluation of temporal summation in individuals with fibromyalgia on long-term opioids. While we had expected individuals on opioids to exhibit enhanced temporal summation, instead, we observed similar temporal summation among all groups. In patients, temporal summation was influenced by chronic pain severity (i.e., ceiling effect) despite our use of sensitivity-adjusted temperatures. Additionally, even though higher pain sensitivity (i.e., lower sensitivity-adjusted temperature) occurred in non-opioid FM, pain sensitivity was similar between healthy controls and opioid-FM, suggesting at least partial thermal opioid analgesia in the opioid-FM group. Meanwhile, within the opioid-FM group, greater thermal pain sensitivity occurred with higher opioid dosage. Thus, as suggested by our results, individuals with fibromyalgia who take opioids do not demonstrate enhanced temporal summation, but they do demonstrate modest thermal analgesia. Further, such individuals on opioid therapy exhibit greater thermal pain sensitivity when taking higher opioid dosages. For the measurement of central sensitization in patients who take opioids, temporal summation relies on complex interactions between chronic pain severity, thermal pain sensitivity, and opioid dosage. Based on our results, future chronic pain research is needed to empirically investigate how opioid use impacts pain sensitivity and central sensitization.

## Data availability statement

The raw data supporting the conclusions of this article will be made available by the authors, without undue reservation.

## Ethics statement

The studies involving humans were approved by the Duke University Institutional Review Board. The studies were conducted in accordance with the local legislation and institutional requirements. The participants provided their written informed consent to participate in this study.

## Author contributions

JB: Formal analysis, Investigation, Methodology, Visualization, Writing – original draft. MR: Formal analysis, Methodology, Software, Writing – review & editing. SP: Validation, Writing – review & editing. AB: Validation, Writing – review & editing. KM: Conceptualization, Funding acquisition, Investigation, Methodology, Project administration, Resources, Supervision, Writing – review & editing.

## Footnotes

^1^OSF, https://doi.org/10.17605/OSF.IO/AQ6TS.
