# Supplementary material for: Interplay between noxious heat sensitivity and temporal summation magnitude in patients with fibromyalgia and long-term opioid use
Source: Front Neurosci. 2023 Oct 12;17:1275921. doi: 10.3389/fnins.2023.1275921 (PMC10600517; doi:10.3389/fnins.2023.1275921)
Supplement: Supplementary file 1 [file Data_Sheet_1.PDF]

Interplay between Noxious Heat Sensitivity and Temporal Summation Magnitude in Patients with Fibromyalgia and Long-term Opioid Use

Jason D Bao; Morgan A Rosser; Su Hyoun Park; Anne K Baker; Katherine T Martucci

Supplementary Material

1 Supplementary Methods

We conducted additional analyses to assess whether temporal summation was influenced by (1) calculation method, (2) opioid phase (i.e., time elapsed since last opioid dose), and (3) stimulus temperature. We also conducted analyses to identify variables potentially associated with temporal summation trajectories.

Variations in baseline pain sensitivities can be better mitigated by calculating temporal summation as a percentage change (Bosma et al, 2018) rather than as a difference calculation (Staud et al, 2001). Thus, we used the "percentage change" calculation method to reevaluate group differences in temporal summation. We also correlated temporal summation, calculated by percentage change, with the psychophysical and opioid use variables from our *a priori* and exploratory analyses, and used the p-values from the original analyses to evaluate significance. A difference calculation was used to evaluate temporal summation in all other supplementary analyses.

Since other clinical measures and demographic variables may influence temporal summation, we conducted additional Spearman correlation analyses to assess the relationship between temporal summation and several other covariates (i.e., age, race, state anxiety, positive affect, total mood disturbance, average pain interference, total pain areas, fatigue, and global symptom severity index). A correlation between variables resulted in a total of 2 independent measures: (1) age (not correlated with other variables) and (2) race, STAI-state anxiety, PANAS positive affect, POMS total mood disturbance, BPI pain interference, FAS total pain areas, PROMIS fatigue, and BSI global severity index (all $p < 0.021$). Therefore, for these correlation analyses, we considered a clinical/demographic measure a significant predictor of temporal summation at $p < 0.025$, correcting for 2 independent comparisons.

The time elapsed since each participant's last opioid dose prior to the study visit could have differentially influenced temporal summation. Thus, based on participant-reported opioid dosing information, we assigned participants to either "early opioid phase" or a "late opioid phase" subgroups to control for differences in opioid dosing patterns. To determine if opioid use patterns were related to heat sensitivity in either subgroup, we analyzed for correlations between each variable related to opioid use (i.e., opioid dosage, duration of opioid use, and time elapsed since last opioid dose) and temporal summation values within each opioid phase subgroup. For this analysis, the significance threshold was established at an uncorrected p-value < 0.05.

As lower noxious stimulus intensity relates to less temporal summation (Nielsen and Arendt-Nielsen, 1998), the use of varied stimulus temperatures (i.e., tailored to individual sensitivity levels) in our study could influence temporal summation across individuals. Therefore, we measured the association between stimulus temperature and temporal summation in our study. First, we first ran a

linear regression analysis among all 3 groups, adjusted for cohort demographic variability (age, ethnicity, race, education, and dominant handedness). Then, controlling for lower VAS = 5 temperatures used in both cohorts of fibromyalgia participants, we reevaluated temporal summation across all participants. For this, we divided non-opioid FM and opioid-FM participants into 2 subgroups: participants with VAS = 5 temperatures ≥ 45°C, and participants with VAS = 5 temperatures < 45°C. We chose to create subgroups with a cut-off temperature of 45°C due to evidence for irreversible protein denaturation and high threshold nociceptor activation at/above this standard noxious temperature (Bischof and He, 2005). After excluding participants with experimental temperatures < 45°C, we ran a one-way ANOVA to compare temporal summation across the subgroups. We set the significance threshold for these analyses at an uncorrected $p < 0.05$.

We then analyzed how rates of temporal summation were related to factors of cohort (non-opioid FM, opioid-FM, and HC), stimulus temperature, and non-opioid medication use. To calculate summation rate (i.e., trajectory, or the rate of change in pain intensity over time), we measured the slope across pain ratings to the 1st, 5th, and 10th stimulus in the series. For each participant, we calculated 2 partial or "sub-slopes" (1st to 5th stimuli; 5th to 10th stimuli) and 1 overall slope (1st to 10th stimuli). We visualized these slope trajectories with ggplot2 (R, version 4.1.3). We used Spearman correlations to assess the relationships of temporal summation slopes with cohort status and stimulus temperature. Additionally, due to analgesic and/or hyperalgesia-reducing properties of non-opioid medications including NSAIDs (Bovill, 1997), SNRIs (Spaeth and Briley, 2009; Obata, 2017), benzodiazepines (Reddy and Patt, 1994), and GABA analogues (Stoicea et al, 2015), we ran exploratory Spearman correlations between the reported mediation use of participants vs. temporal summation slopes. We set the significance threshold for all temporal summation slope correlations at an uncorrected p-value < 0.05.

Finally, because 3 different female experimenters collected the data for this study, we ran a one-way ANOVA to compare temporal summation measurements among all experimenters, within each participant group. Additional post-hoc *t*-tests were conducted between experimenters upon significant ANOVA results ($p < 0.05$).

2 Supplementary Results

2.1 Reevaluation of *a priori* and Exploratory Analyses

For all *a priori* and exploratory analyses (i.e., comparison of temporal summation across groups, association between temporal summation and psychophysical variables, and association between temporal summation and opioid use variables), we reevaluated the original difference change calculation results as compared to a percentage change calculation for temporal summation.

Our reevaluated results largely mirrored our primary findings. Using a one-way ANOVA to compare temporal summation across all participant groups, we did not find significant differences in summation ($F_{(2, 85)} = 0.369$, $p = 0.693$). Similarly, among the psychophysical variables, only BPI pain severity significantly correlated with temporal summation (r = -0.36, $p = 0.006$, n = 55). All tested correlations of temporal summation with psychophysical variables are displayed in Supplementary Table 1.

Further, temporal summation did not significantly correlate with opioid dosage nor duration of use (dosage: r = -0.09, $p = 0.685$, n = 22; duration of use: r = 0.27, $p = 0.236$, n = 21). However, we identified a trending correlation between temporal summation and time elapsed since last opioid dose (r = 0.49, $p = 0.026$, n = 21) that did not survive Bonferroni correction.

Supplementary Table 1. Tested Variables for Correlation with Temporal Summation (Percentage Change). Spearman correlations across all participants reevaluated the relationship between clinical, psychological, behavioral, and demographic variables, and temporal summation, calculated as a percentage change in perceived pain. Correlation tests were conducted within each fibromyalgia subgroup – non-opioid FM and opioid-FM participants. Comparisons differed in the number of data values (± 5) due to some incomplete questionnaires during the study visit. Similar to the primary analyses, we observed a significant correlation between pain severity and reduced temporal summation in the fibromyalgia groups. Healthy controls did not complete the BPI questionnaire. Significance was evaluated at a Bonferroni-corrected p-value < 0.025 for both *a priori* and exploratory variables. **p* < 0.025.

Variable	TS - All Participants (N = 88)		TS - FM (n = 57)		TS - Non-opioid FM (n = 33)		TS - Opioid-FM (n = 24)	
	Rho	P-Value	Rho	P-Value	Rho	P-Value	Rho	P-Value
Trait Anxiety (STAI)	-0.07	0.559	-0.04	0.754	-0.08	0.685	0.01	0.971
Depression (BDI)	0.05	0.654	0.15	0.277	0.21	0.236	0.02	0.945
Pain Severity (BPI)	-	-	-0.36	0.006*	-0.06	0.736	-0.41	0.049
Negative Affect (PANAS)	-0.03	0.763	-0.08	0.548	0.05	0.779	-0.22	0.304

2.2 Temporal Summation Correlations with Additional Clinical and Demographic Variables

Across the 3 participant groups, temporal summation was not significantly correlated with the exploratory variables (age, race, STAI-state anxiety, PANAS positive affect, POMS total mood disturbance, BPI pain interference, FAS total pain areas, PROMIS fatigue, and BSI global severity index) (all corrected for multiple comparisons). However, among the opioid-FM participants, temporal summation trended toward a negative correlation with age ($r = –0.36$, $p = 0.041$, $n = 24$), which could reflect reduced pain responsiveness with greater age in individuals taking opioids. While this finding adds to the growing literature of age differences in forearm heat pain sensitivity (Farrell and Gibson, 2007; Lautenbacher, 2012; Riley et al, 2019; Daguet et al, 2020), it does not provide evidence for dysfunctional pain inhibition with age. Rather, this finding may be due to the complex interactions between opioids and the altered pain processing in individuals with fibromyalgia. Further, across the fibromyalgia groups, pain severity significantly correlated with state anxiety ($r = 0.47$, $p < 0.001$, $n = 54$). Results for the additional correlations are shown in Supplementary Table 2.

Supplementary Table 2. Additional Variables That Did Not Correlate with Temporal Summation. Using Spearman correlation tests across all 3 groups, we assessed potential influences of exploratory clinical, psychological, behavioral, and demographic variables on temporal summation. We also measured correlations within each fibromyalgia subgroup (i.e., non-opioid FM, and opioid-FM). As some of the questionnaires were not completed during the study visit, the number of included participants for each test varied (± 5). No significant correlations were found. Healthy controls did not complete the BPI questionnaire. Significance was evaluated at a Bonferroni-corrected p-value < 0.025 for both *a priori* and exploratory variables.

Variable	TS - All Participants (N = 88)		TS – FM (n = 57)		TS - Non-opioid FM (n = 33)		TS - Opioid-FM (n = 24)	
	Rho	P-Value	Rho	P-Value	Rho	P-Value	Rho	P-Value
**Age**	-0.16	0.073	-0.13	0.172	0.09	0.688	-0.36	0.041
**Race**	0.07	0.509	0.12	0.385	0.16	0.363	0.04	0.857
**State Anxiety (STAI)**	-0.06	0.561	-0.14	0.293	-0.16	0.387	0.06	0.800
**Positive Affect (PANAS)**	0.04	0.712	0.18	0.186	0.13	0.473	0.26	0.216
**Total Mood Disturbance (POMS)**	-0.01	0.957	-0.16	0.246	-0.18	0.327	-0.18	0.403
**Pain Interference (BPI)**	-0.14	0.298	-0.14	0.298	-0.17	0.367	-0.15	0.472
**Total Pain Areas (FAS)**	0.02	0.863	-0.05	0.701	-0.01	0.978	-0.08	0.723
**Fatigue (PROMIS)**	0.04	0.724	-0.02	0.911	0.03	0.888	-0.08	0.726
**Global Severity Index (BSI)**	-0.03	0.793	-0.24	0.076	-0.28	0.116	-0.41	0.045

2.3 Opioid Usage

Based on the self-reported time of their last opioid dose prior to the study visit, opioid-FM participants were divided into 2 subgroups that corresponded to "early phase" and "late phase" of opioid dosing at the time of temporal summation testing. For our defining criteria for the "early phase" subgroup, participants underwent temporal summation testing within the first or second half-life of their opioid medication. For our defining criteria for the "late phase" subgroup, participants underwent temporal summation testing within the third half-life or later of their opioid medication. Based on these subgroup definitions, we identified 9 participants who had temporal summation tested in the "early phase" of opioid dosing, and 15 participants who were tested in the "late phase" of opioid dosing (Supplementary Figure 1). To determine opioid phase, we calculated the approximate metabolism rate for each specific opioid medication, based on FDA or package insert sheet data. For any participants who were taking multiple opioid medications, we used the opioid medication with the longer half-life to determine their opioid phase subgroup assignment. Further information regarding the variations in opioid dosage and average duration of opioid use are displayed in Supplementary Table 3.

Overall, within each opioid phase subgroup (i.e., early phase and late phase), temporal summation did not significantly correlate with any of the 3 opioid variables (i.e., opioid dosage, duration of opioid use, and time elapsed since last opioid dose). All correlations are shown in Supplementary Table 4.

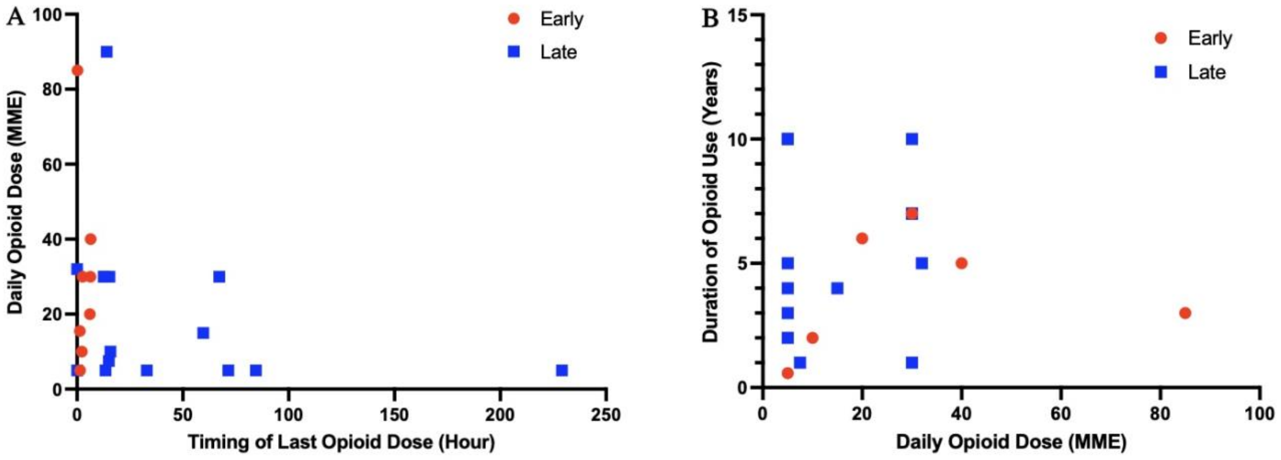

Supplementary Figure 1. Opioid Consumption Timing, Dosage, and Duration of Use. Early and late phase opioid-FM participants are differentiated by color. As defined for early phase participants, temporal summation was tested during opioid first or second half-life. As defined for late phase participants, temporal summation was tested during opioid third half-life or later. (A) Daily opioid dose, calculated as morphine milligram equivalents (MME) vs time elapsed since last opioid dose in hours. The 2 variables were negatively correlated (r = -0.427, p = 0.030), indicating that participants with higher dosage opioid prescriptions consumed them more frequently in our cohort. One outlier late phase participant had a last opioid dose 233.5 hours before the study visit. Two participants did not specify the exact time of their last opioid dose, and were not included in the graph; however, they reported most recent opioid use 9 days and 1 month before the study visit, respectively. (B) Daily opioid dose vs duration of opioid use in years. Dosage and duration of opioid use were not significantly correlated (r = 0.172, p = 0.469). Two opioid-FM participants, one early phase and one late phase, had similar self-reported daily opioid dosages and durations of opioid usage, resulting in overlapping points in the plot. Three participants did not report their duration of opioid use, and one participant did not report opioid dosage.

Supplementary Table 3. Range of Dosage and Duration of Use per Type of Opioid. The range of daily opioid dosage, calculated as morphine milligram equivalents (MME), and average duration of opioid use in years for each type of opioid medication are listed. Codeine, hydromorphone, methadone, and oxycodone, and oxycodone/acetaminophen were each taken by 1 opioid-FM participant prior to the study visit. *One participant did not report opioid dosage (1 opioid-FM taking codeine). ^3 participants did not report their duration of opioid use (1 opioid-FM taking hydrocodone/acetaminophen, oxycodone/acetaminophen, and tapentadol, respectively).

Opioid Type	Range of MME Dosage	Average Duration of Use (Years)
**Codeine**	*	15
**Hydrocodone**	7.5 - 15	1
**Hydrocodone/acetaminophen (eg, Norco)**	5 - 40	6.2^
**Hydromorphone**	8	5
**Methadone**	20	6
**Morphine ER**	30 - 45	4
**Oxycodone**	30	10
**Oxycodone/acetaminophen (eg, Percocet)**	15.5	^
**Tapentadol (eg, Nucynta)**	30 - 90	1^
**Tramadol**	5 - 30	4.7

Supplementary Table 4. No Significant Correlations Between Temporal Summation and Opioid Use Patterns for Participants Tested during the Early vs Late Opioid Phase. After defining the "early" and "late" phase subgroups of opioid-FM participants, patterns of opioid use were tested for correlations with temporal summation. One participant did not demonstrate any temporal summation; one participant did not report daily dosage; 2 participants did not report duration of use; and one participant did not report timing of last dose. Spearman correlations were assessed for significance at an uncorrected threshold of $p < 0.05$.

Opioid Variable	Temporal Summation Early Phase			Temporal Summation Late Phase		
	**n**	**Rho**	**P-value**	**n**	**Rho**	**P-value**
**Opioid Dosage**	7	-0.23	0.613	14	0.50	0.066
**Duration of Opioid Use**	8	0.66	0.076	12	0.07	0.835
**Timing of Last Dose**	8	0.52	0.197	12	-0.15	0.676

2.4 Relationships between Stimulus Temperature and Temporal Summation

As identified through a univariate regression analysis, stimulus temperature was significantly associated with temporal summation, such that temporal summation increased by 1.84 (95% CI: [0.03, 3.66]) with each unit increase in stimulus temperature ($F_{(2, 86)} = 4.073$, $p = 0.047$). This indicated that stimulus temperature may have had an impact on temporal summation. However, after adjusting for age, ethnicity, race, education, and handedness in a multivariate model, the association was no longer significant ($p = 0.209$).

When re-examining temporal summation across all 3 groups after excluding participants with VAS = 5 temperatures < 45°C, we identified no significant group differences in temporal summation ($F_{(2, 69)} = 0.265$, $p = 0.768$). This re-examined result was similar to the results from our main analysis (which included all participants with all sensitivity-adjusted stimulus temperatures). Thus, regardless of actual stimulus temperature used, when given similar intensity noxious stimuli (i.e., sensitivity-adjusted temperatures to VAS = 5), all participants (non-opioid FM, opioid-FM, and HC) demonstrated comparable temporal summation.

2.5 Factors Associated with Temporal Summation Slopes

The temporal summation slopes (ie, rates of change/summation; VAS pain rating ratios) of all participants, calculated from pain intensity ratings after the 1st, 5th, and 10th stimuli, are shown in Supplementary Figure 2. For sub-slopes measured between the 1st and 5th stimuli, non-opioid FM participants demonstrated the steepest slopes (0.29), compared to opioid-FM (0.22) and HC (0.25) participants. For sub-slopes measured between the 5th and 10th stimuli, all groups were similar (0.23, 0.22, and 0.21 for non-opioid FM, opioid-FM, and HC, respectively). On average, while the opioid-FM group displayed more consistent summation rates across all 10 stimuli, both non-opioid FM and HC groups showed reduced summation rates after the first 5 stimuli. Correlations between slopes and stimulus temperature did not reach significance across the 3 participant groups nor across the fibromyalgia groups (Supplementary Table 5). Although all 3 groups exhibited heterogeneity in slopes, the extent to which these differences can be attributed to stimulus temperature remained unclear.

As measured by Spearman correlations across all participants, we did not observe any significant relationships for slopes with patient reported NSAID, SNRI, benzodiazepine, and GABA analogue usage. Comparisons within each group also did not reach significance. All correlations are displayed in Supplementary Table 6.

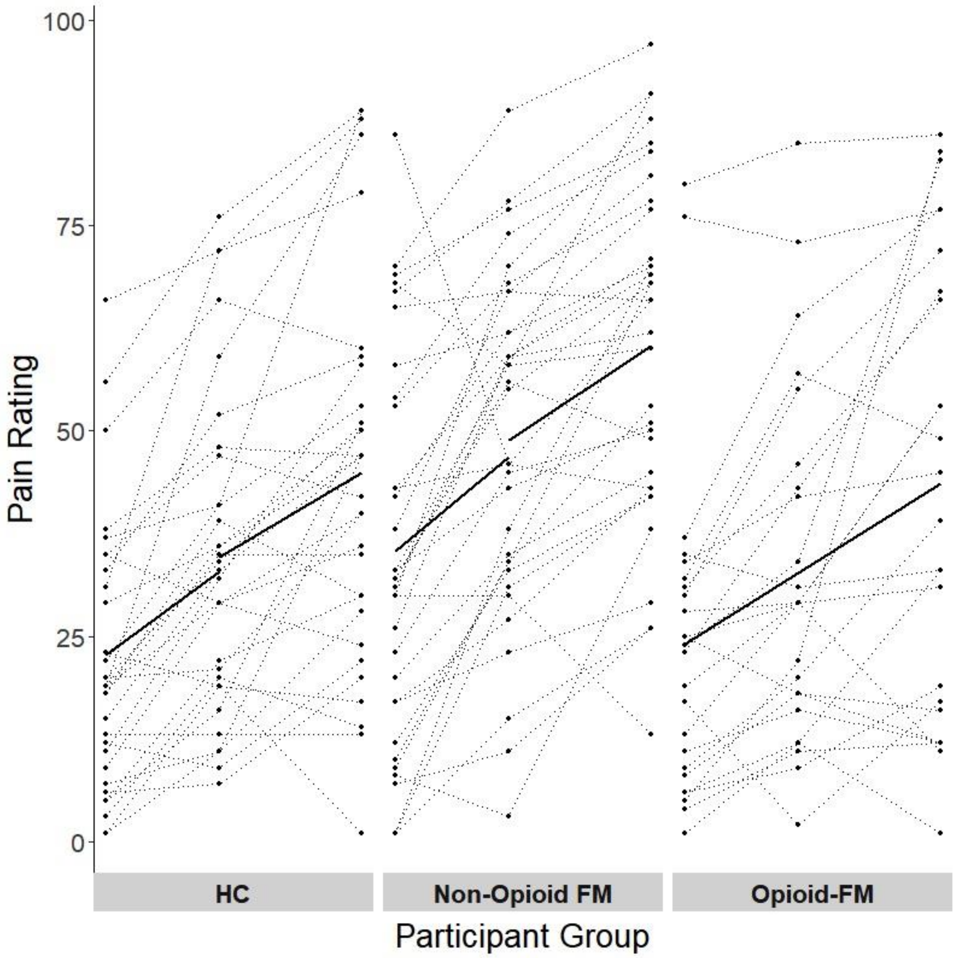

Supplementary Figure 2. Slopes of Temporal Summation to Noxious Thermal Stimuli. The pain ratings after the 1st, 5th, and 10th stimulus were used to determine the rates of summation during the first and second halves of the temporal summation paradigm. The plotted points and dotted lines represent participant-specific ratings and slopes, and the bolded line represents the mean slope per group. From the 1st to 5th stimuli, the non-opioid FM group had the steepest slope, with pain ratings increasing by 0.29 units per stimulus. Opioid-FM and HC groups showed less steep slopes of 0.22 and 0.25, respectively. From the 5th to 10th stimuli, the non-opioid FM group had the steepest slope at 0.23 units per stimulus, compared to opioid-FM (0.22) and HC (0.21) participants.

Supplementary Table 5. Stimulus Temperature vs Temporal Summation Slopes. The association between stimulus temperature (the VAS = 5 temperature) and temporal summation slopes was assessed for all participants, and then separately across both fibromyalgia groups, to limit control bias. Slopes were determined based on rate of change in pain responses between the 1st and 5th stimuli, 5th and 10th stimuli, and 1st and 10th stimuli. No Spearman correlations were significant at an uncorrected p-value < 0.05.

	Slope	Stimuli Temperature		
		n	Rho	P-Value
All Participants	Stimuli 1 - 5	88	0.07	0.508
	Stimuli 5 - 10	88	0.21	0.053
	Stimuli 1 - 10	88	0.12	0.264
Non-opioid FM + Opioid-FM	Stimuli 1 - 5	57	0.09	0.494
	Stimuli 5 - 10	57	0.23	0.091
	Stimuli 1 - 10	57	0.15	0.271

Supplementary Table 6. Supplementary Drug Use and Temporal Summation Slopes. Spearman correlations between non-opioid pain-related medication use and temporal summation slope across all participants and within each participant group are reported. Slopes were measured in terms of the 1st, 5th, and 10th stimuli, and were subsequently evaluated against use of each reported non-opioid medication. Among healthy control participants, 2 were taking NSAIDs, and none reported use of SNRIs, benzodiazepines, or GABA analogues. Among non-opioid FM participants, 8 were taking NSAIDs, 10 were taking SNRIs, 7 were taking benzodiazepines, and 5 were taking GABA analogues. Among opioid-FM participants, 8 were taking NSAIDs, 9 were taking SNRIs, 3 were taking benzodiazepines, and 9 were taking GABA analogues. No significant correlations were observed at an uncorrected threshold p-value < 0.05. Abbreviations: NSAID, nonsteroidal anti-inflammatory drug; SNRI, serotonin and noradrenergic reuptake inhibitor; GABA, gamma-aminobutyric acid.

	Slope	NSAID		SNRI		Benzodiazepine		GABA Analogue	
		Rho	P-Value	Rho	P-Value	Rho	P-Value	Rho	P-Value
All Participants	Stimuli 1 - 5	0.01	0.925	0.07	0.532	0.07	0.544	-0.07	0.533
	Stimuli 5 - 10	-0.08	0.434	0.17	0.121	0.04	0.696	0.10	0.373
	Stimuli 1 - 10	-0.03	0.805	0.12	0.271	0.06	0.602	0.06	0.564
HC	Stimuli 1 - 5	0.26	0.163	-	-	-	-	-	-
	Stimuli 5 - 10	0.16	0.385	-	-	-	-	-	-
	Stimuli 1 - 10	0.29	0.109	-	-	-	-	-	-
Non-opioid FM	Stimuli 1 - 5	0.07	0.712	0.29	0.100	-0.08	0.651	0	0.980
	Stimuli 5 - 10	-0.27	0.132	0.27	0.128	-0.08	0.651	0.03	0.883
	Stimuli 1 - 10	-0.03	0.853	0.29	0.100	-0.16	0.387	0.02	0.902
Opioid-FM	Stimuli 1 - 5	-0.17	0.439	-0.10	0.643	0.22	0.305	-0.07	0.729
	Stimuli 5 - 10	-0.08	0.712	0.16	0.450	0.25	0.247	0.24	0.254
	Stimuli 1 - 10	-0.18	0.403	-0.04	0.840	0.34	0.108	0.11	0.623

2.6 Similar Between-Experimenter Temporal Summation Measurements

Three female experimenters collected the data for this study: Data from the HC group was collected by 2 of the experimenters, while data from the non-opioid FM and opioid-FM groups were collected by 3 of the experimenters. When comparing measurements of temporal summation as collected by the different experimenters, temporal summation was not significantly different for any group (HC: $F_{(1, 29)} = 1.991$, $p = 0.169$; non-opioid FM: $F_{(2, 30)} = 2.386$, $p = 0.109$; opioid-FM: $F_{(2, 21)} = 0.286$, $p = 0.754$).

Supplementary Material References:

Bischof, J.C., and He, X. (2005). Thermal stability of proteins. *Ann N Y Acad Sci* 1066**,** 12-33. doi: 10.1196/annals.1363.003.

Bosma, R.L., Cheng, J.C., Rogachov, A., Kim, J.A., Hemington, K.S., Osborne, N.R., et al. (2018). Brain Dynamics and Temporal Summation of Pain Predicts Neuropathic Pain Relief from Ketamine Infusion. *Anesthesiology* 129(5)**,** 1015-1024. doi: 10.1097/aln.0000000000002417.

Bovill, J.G. (1997). Mechanisms of actions of opioids and non-steroidal anti-inflammatory drugs. *Eur J Anaesthesiol Suppl* 15**,** 9-15. doi: 10.1097/00003643-199705001-00003.

Daguet, I., Bergeron-Vezina, K., Harvey, M.P., Martel, M., Coulombe-Leveque, A., and Leonard, G. (2020). Decreased Initial Peak Pain Sensation with Aging: A Psychophysical Study. *J Pain Res* 13**,** 2333-2341. doi: 10.2147/jpr.S257791.

Farrell, M., and Gibson, S. (2007). Age interacts with stimulus frequency in the temporal summation of pain. *Pain Med* 8(6)**,** 514-520. doi: 10.1111/j.1526-4637.2007.00282.x.

Lautenbacher, S. (2012). Experimental approaches in the study of pain in the elderly. *Pain Med* 13 Suppl 2**,** S44-50. doi: 10.1111/j.1526-4637.2012.01326.x.

Nielsen, J., and Arendt-Nielsen, L. (1998). The importance of stimulus configuration for temporal summation of first and second pain to repeated heat stimuli. *Eur J Pain* 2(4)**,** 329-341. doi: 10.1016/s1090-3801(98)90031-3.

Obata, H. (2017). Analgesic Mechanisms of Antidepressants for Neuropathic Pain. *Int J Mol Sci* 18(11). doi: 10.3390/ijms18112483.

Reddy, S., and Patt, R.B. (1994). The benzodiazepines as adjuvant analgesics. *J Pain Symptom Manage* 9(8)**,** 510-514. doi: 10.1016/0885-3924(94)90112-0.

Riley, J.L., 3rd, Cruz-Almeida, Y., Staud, R., and Fillingim, R.B. (2019). Effects of manipulating the interstimulus interval on heat-evoked temporal summation of second pain across the age span. *Pain* 160(1)**,** 95-101. doi: 10.1097/j.pain.0000000000001382.

Spaeth, M., and Briley, M. (2009). Fibromyalgia: a complex syndrome requiring a multidisciplinary approach. *Hum Psychopharmacol* 24 Suppl 1**,** S3-10. doi: 10.1002/hup.1030.

Staud, R., Vierck, C.J., Cannon, R.L., Mauderli, A.P., and Price, D.D. (2001). Abnormal sensitization and temporal summation of second pain (wind-up) in patients with fibromyalgia syndrome. *PAIN* 91(1).

Stoicea, N., Russell, D., Weidner, G., Durda, M., Joseph, N.C., Yu, J., et al. (2015). Opioid-induced hyperalgesia in chronic pain patients and the mitigating effects of gabapentin. *Front Pharmacol* 6**,** 104. doi: 10.3389/fphar.2015.00104.
